# Stay-at-Home Orders during COVID-19: The Influence on Physical Activity and Recreational Screen Time Change among Diverse Emerging Adults and Future Implications for Health Promotion and the Prevention of Widening Health Disparities

**DOI:** 10.3390/ijerph182413228

**Published:** 2021-12-15

**Authors:** Daheia J. Barr-Anderson, Vivienne M. Hazzard, Samantha L. Hahn, Amanda L. Folk, Brooke E. Wagner, Dianne Neumark-Sztainer

**Affiliations:** 1 School of Kinesiology, University of Minnesota, Minneapolis, MN 55455, USA; folk0039@umn.edu (A.L.F.); wagn0900@umn.edu (B.E.W.); 2 Department of Psychiatry & Behavioral Health, University of Minnesota, Minneapolis, MN 55455, USA; vhazzard@umn.edu (V.M.H.); hahn0203@umn.edu (S.L.H.); 3 Division of Epidemiology and Community Health, University of Minnesota, Minneapolis, MN 55455, USA; neumark@epi.umn.edu

**Keywords:** sedentary behavior, physical activity, screen time, pandemic, longitudinal study

## Abstract

Background: The purpose of this study was to examine changes in physical activity (PA) and recreational screen time (RST) behaviors from pre-COVID-19 in 2018 to Spring 2020 during the mandatory stay-at-home order in an ethnically/racially, socioeconomically diverse sample of emerging adults. Methods: Longitudinal data were analyzed from 218 participants (*M*_age_ = 24.6 ± 2.0 years) who completed two surveys: EAT 2018 (Eating and Activity over Time) and C-EAT in 2020 (during COVID-19). Repeated ANCOVAs and multiple linear regression models were conducted. Results: Moderate-to-vigorous and total PA decreased (4.7 ± 0.3 to 3.5 ± 0.3 h/week [*p* < 0.001] and 7.9 ± 0.4 to 5.8 ± 0.4 h/week [*p* < 0.001], respectively), and RST increased from 26.5 ± 0.9 to 29.4 ± 0.8 h/week (*p* = 0.003). Perceived lack of neighborhood safety, ethnic/racial minoritized identities, and low socioeconomic status were significant predictors of lower PA and higher RST during COVID-19. For example, low SES was associated with 4.04 fewer hours of total PA compared to high SES (*p* < 0.001). Conclusions: Stay-at-home policies may have significantly influenced PA and RST levels in emerging adults with pre-existing disparities exacerbated during this mandatory period of sheltering-in-place. This suggests that the pandemic may have played a role in introducing or magnifying these disparities. Post-pandemic interventions will be needed to reverse trends in PA and RST, with a focus on improving neighborhood safety and meeting the needs of low socioeconomic and ethnic/racial minoritized groups.

## 1. Introduction

Prior to the worldwide pandemic of coronavirus disease 2019 (COVID-19), inadequate physical activity (PA) and excessive screen time were already public health concerns for people living in the United States, as physical inactivity and sedentary behavior are highly prevalent risk factors for cardiovascular disease, obesity, cancer, diabetes, hypertension, depression, and premature death [1]. As a result of the stay-at-home orders implemented both domestically and internationally to curb the spread of COVID-19, public venues such as fitness centers, certain parks, schools, restaurants, and theaters closed [2], and the public was encouraged to stay at home as much as possible and only leave for essential activities.

As many people were urged to stay within the confines of their homes and faced with the cancellation of in-person activities (i.e., on-site work, school, extracurricular activities), important health behaviors, such as PA and screen time, may have been affected. The unprecedented social disruption of COVID-19 introduced unique barriers to regular movement, including closure of spaces and activities dedicated to PA [3], while encouraging more screen time for educational, occupational, and recreational use [4]. It is likely that because people were unable to spend time outside of their homes engaging in their usual PA, they were more sedentary and consumed more screen time because of boredom, social isolation, and to stay informed about COVID-19 [3,4]. Because of the potential influence of the stay-at-home orders on PA and screen time behavior, there is a need to quantify the impact of COVID-19 on these health behaviors.

One population of particular concern is emerging adults aged 18 to 29 years, particularly those from lower socioeconomic and ethnically/racially diverse backgrounds. Emerging adulthood is a period where individuals begin to explore and make their own decisions independent of family, friends, and school [5]; this includes decisions related to PA and screen time behaviors. Further, this population has been heavily affected by the pandemic, particularly in regards to mental health [6]. It is well established that PA and screen time are both strongly associated with mental and physical health [7]. Therefore, realizing the potential implications of COVID-19 on PA and screen time will help us better understand the prospective long-term effects of the pandemic and inform public health interventions aimed at improving health outcomes for this population.

Given the influence of PA and screen time on health and the importance of maintaining health during the critical developmental period of emerging adulthood, the purpose of this study is to examine changes in PA and recreational screen time from pre-COVID-19 in 2018 to during the Spring 2020 COVID-19 mandatory stay-at-home order in an ethnically/racially and socioeconomically diverse sample of emerging adults. Additionally, we explore sociodemographic and neighborhood safety predictors to better understand who may be at an increased risk of decreased PA and increased recreational screen time. Findings have implications for understanding the impact of stay-at-home policies on the overall health and well-being of populations to inform future policies and health interventions.

## 2. Materials and Methods

### 2.1. Study Design and Sample

The EAT 2010–2018 (Eating and Activity over Time) study is a population-based, longitudinal investigation of weight-related health behaviors and associated factors among young people who attended secondary school in Minneapolis-St. Paul, Minnesota [8,9]. Participants were initially recruited in 2009–2010 and completed an EAT 2010 survey at that time. The sample for the present study was comprised of EAT 2010 participants who subsequently completed the EAT 2018 survey, which was administered in 2017–2018 (pre-pandemic), as well as the C-EAT survey, which was administered during the initial months of the COVID-19 outbreak [3,4,10,11,12,13,14]. Invitations to participate were sent to cohort members by email and text message in April 2020. Given the purpose of the current study, in which we wanted to examine the impact of stay-at-home orders, only surveys that were completed and returned by 18 May 2020, the date that the initial mandatory stay-at-home order was lifted in Minnesota, were included in the analyses (N = 218). All participants were mailed a financial incentive following survey completion. The University of Minnesota Institutional Review Board Human Subjects Committee approved all protocols, and informed consent was obtained from all participants in the study.

### 2.2. Measures

The C-EAT survey was based on prior EAT surveys [15,16], with modifications made to focus recall on the past month of events related to the COVID-19 outbreak. Information was included with the survey to inform participants that the goal of the survey was to learn how their experiences with the COVID-19 outbreak may be influencing their eating behaviors and well-being. Test-retest reliability of measures was examined at EAT 2018 using data from 112 participants who completed the survey twice over 3 weeks; these estimates are provided below for each measure.

#### 2.2.1. Physical Activity

Hours of past-week strenuous, moderate, and light PA were assessed in the EAT 2018 and C-EAT surveys using items modified from the Godin-Shephard Leisure-Time Exercise Questionnaire [17] and validated against accelerometry data in a subsample of young adult participants in an earlier EAT cohort [18]. Each item included relevant exercise examples for strenuous exercise (biking fast, aerobics, jogging, basketball, swimming laps, soccer, rollerblading), moderate exercise (walking quickly, easy bicycling, volleyball, skiing, dancing, skateboarding, snowboarding), and light exercise (walking slowly, bowling, golf, fishing, snowmobiling). Response options ranged from “none” to “6+ h a week”. The midpoint of each of the six response options was used to calculate hours per week spent in each intensity category, with the “6+” category counted as 8 h per week. Moderate-to-vigorous PA (MVPA) was calculated as the sum of hours spent in strenuous and moderate exercise per week (Spearman rank-order test-retest correlation *r* = 0.72), and total PA was calculated as the sum of hours spent in strenuous, moderate, and mild exercise per week (Pearson product-moment test-retest correlation *r* = 0.66).

#### 2.2.2. Recreational Screen Time

In the EAT 2018 and C-EAT surveys, participants were asked to report how many hours per day of recreational screen time (e.g., television, computer, social media, video games, smartphone or tablet; excluding activities for work or school) they engaged in on an average weekday and on an average weekend day. Measures were modified from prior EAT surveys [19,20] to be relevant to current technology. On the C-EAT survey, these items specifically asked participants to report on the past month; no time period was specified on the EAT 2018 survey. Seven response options ranged from “0 h a day” to “5+ h a day.” The value of each response option was used to calculate hours per day engaged in recreational screen time on weekdays and weekend days, with “5+ h a day” counted as 6 h per day. These values were summed (average weekday*5 + average weekend*2) to calculate total hours per week of recreational screen time (test-retest Pearson product-moment correlation *r* = 0.76) [19,20].

#### 2.2.3. Perceived Lack of Neighborhood Safety

To assess perceived lack of neighborhood safety in the C-EAT survey, participants responded *yes* or *no* to the following items from the Neighborhood Environment Walkability Scale: “I feel safe walking in my neighborhood during the day” and “I feel safe walking in my neighborhood at night” (test-retest *r* = 0.82) [21,22].

#### 2.2.4. Sociodemographic Variables

Structurally racialized categories labeled as ethnicity/race and several indicators of socioeconomic status (SES) were self-reported in the EAT 2010 survey. Ethnicity/race was assessed by self-report with the question: “Do you think of yourself as…? (You may choose more than one)”, with the following response options: White, Black or African American, Hispanic or Latino, Asian American, Native Hawaiian or Pacific Islander, American Indian or Native American, or Other. Participants who selected both “Hispanic or Latino” and “White” were categorized as Hispanic/Latinx for analysis. Additionally, participants who reported more than one race, or reported Hispanic/Latinx ethnicity and any race other than White, were coded as “mixed” and combined with participants reporting “other” for analysis. Since very few participants (n = 2) reported “Hawaiian or Pacific Islander” or did not respond, these participants were also included in the category “mixed or other.”

A five-category SES variable [23] was primarily determined by the highest education level of either participant’s parent (did not finish school, finished high school or GED, some college, finished college, master’s or doctoral degree, do not know). An algorithm including the following additional variables was used to reduce the impact of missing data and to prevent SES misclassification: family eligibility for public assistance (yes, no, do not know), adolescent eligibility for free or reduced-price school lunch (yes, no, do not know), and maternal and paternal employment status (full-time, part-time, not working, do not know). More specifically, this algorithm did not classify youth as high SES solely on parental education and utilized Classification and Regression Trees developed by Brieman et al. [24] to show that the other variables predicted parental education and reduced the level of missing SES values. Age in years at each time-point was calculated by subtracting the participant’s birthdate from each survey completion date. Additional sociodemographic characteristics were assessed in the C-EAT survey: participants reported gender (male/female/other), number of children in their household, and whether or not they were temporarily laid off due to the COVID-19 situation with no other current work.

### 2.3. Statistical Analysis

Repeated ANCOVA analyses were conducted to examine changes in MVPA, total PA, and recreational screen time pre-pandemic (EAT 2018) through the pandemic stay-at-home orders (C-EAT). Analyses controlled for the month of the year during which the EAT 2018 survey was completed to account for potential seasonal differences. The percentages of participants who experienced decreases, no changes, or increases in MVPA, total PA, and recreational screen time from pre-pandemic through the pandemic stay-at-orders were also calculated and presented as descriptive statistics. Linear regression models were used to examine if perceived lack of neighborhood safety and sociodemographic factors predicted MVPA, total PA, and recreational screen time through the pandemic stay-at-home orders. All models adjusted for pre-pandemic levels of the appropriate outcome as assessed at EAT 2018 (e.g., models predicting total PA through the pandemic stay-at-home orders were adjusted for pre-pandemic total PA). Perceived lack of neighborhood safety and sociodemographic factors were initially examined in separate models. Perceived lack of neighborhood safety and sociodemographic predictors were subsequently examined in mutually adjusted models, which included all sociodemographic factors, and whichever perceived lack of neighborhood safety variable (i.e., daytime or nighttime) was found to be a stronger predictor in separate models for each respective outcome. SAS 9.4 (SAS Institute, Cary, NC, USA) was used to complete data analyses.

## 3. Results

### 3.1. Descriptive Analyses

Participants of this study (N = 218 emerging adults) were ethnically/racially and socioeconomically diverse and predominantly identified as female (Table 1). During the initial stay-at-home orders of the pandemic, 11.5% and 40.8% of participants reported feeling unsafe walking in their neighborhood during the day and night, respectively. Approximately 20% of participants were temporarily laid off due to COVID-19, and 20% had children at home.

### 3.2. Changes in Amount of PA and Recreational Screen Time

From pre-pandemic through the pandemic stay-at-home orders, on weekly average, MVPA decreased from 4.7 to 3.5 h (*p* < 0.001), total PA decreased from 7.9 to 5.8 h (*p* < 0.001), and recreational screen time increased from 26.5 to 29.4 h (*p* = 0.003) (Table 2). These results were adjusted for the month in which the EAT 2018 survey was completed, suggesting the observed differences were not explained by seasonal fluctuations in pre-pandemic PA and screen time. Although the majority of the emerging adults reported decreases in MVPA (60.4%) and total PA (58.8%), a substantial number of participants reported increases in MVPA and total PA (30.4% and 32.4%, respectively) (Table 3). Few participants reported no change in MVPA (9.2%) or total PA (8.8%). Slightly less than half (48.6%) of the sample reported an increase in recreational screen time, while 29.6% reported a decrease in recreational screen time, and 21.8% reported no change.

### 3.3. Predictors of PA and Recreational Screen Time

After adjusting for pre-pandemic levels of the outcome, several significant predictors of PA and recreational screen time during the pandemic stay-at-home order were identified (Table 4). Perceived lack of neighborhood safety during the day was associated with lower MVPA and total PA, while perceived lack of neighborhood safety at night was associated with greater recreational screen time. Compared to white participants, Black or African American participants, Asian American participants, and participants of mixed or other ethnic/racial categories reported lower levels of MVPA and total PA, while Asian American participants reported higher levels of recreational screen time. Additionally, compared to participants of high SES, participants of low, low-middle, and middle SES reported lower levels of MVPA and total PA, and participants of high-middle SES reported lower levels of total PA. For example, participants of low SES reported, on average, 4.04 fewer hours of total PA per week during the pandemic stay-at-home order compared to participants of high SES.

In mutually adjusted models, a number of predictors of PA and recreational screen time during the pandemic stay-at-home order remained significant (Table 5). For example, perceived lack of neighborhood safety during the day was associated with, on average, 2.75 fewer hours of total PA per week, and perceived lack of neighborhood safety at night was associated with, on average, 3.56 more hours of recreational screen time per week. Compared to white participants, Black or African American participants and participants of mixed or other ethnic/racial categories reported lower levels of MVPA and total PA during the pandemic stay-at-home order, while participants identifying as Asian American reported higher levels of recreational screen time during COVID-19. Additionally, compared to participants of high SES, participants of low SES reported lower levels of MVPA and total PA during the pandemic stay-at-home order.

## 4. Discussion

The purpose of this study was to examine the change in PA behavior and recreational screen time from 2018 (pre-pandemic) to Spring 2020 (during the COVID-19 mandatory stay-at-home order) in an ethnically/racially and socioeconomically diverse sample of emerging adults. After accounting for pre-pandemic seasonal fluctuations, on average, PA levels decreased, and recreational screen time use increased. However, when examining individual change in PA and recreational screen time, there was variation, with almost a third of the sample reporting being more physically active and engaging in less recreational screen time during the COVID-19 mandatory stay-at-home order compared to pre-pandemic. More in-depth analyses revealed perceived lack of neighborhood safety during the day where Black or African American, Asian American, mixed or other ethnicities/races, or coming from a lower socioeconomic background were associated with lower MVPA and total PA during the COVID-19 mandatory stay-at-home order after adjusting for pre-pandemic PA levels. Perceived lack of neighborhood safety at night and being Asian American were associated with greater recreational screen time during this same period. These findings raise concerns about critical health behaviors among subgroups of the population already at higher risk for adverse health outcomes.

Although numerous U.S. and international studies have been published examining PA and screen time during COVID-19, very few investigations have examined changes in these behaviors related to the mandatory stay-at-home orders. Most studies have examined cross-sectional, rather than longitudinal, associations. Four studies have reported longitudinal changes, similar to the current study [25,26,27,28], with all but one of them [25] reporting similar findings. A sample of 112 middle-aged, majority-white participants who were fully employed during COVID-19 mandatory stay-at-home orders reported no change in PA but an increase in screen time. Increased screen time could be explained by participants shifting from in-person to remote work; however, the authors did not provide an explanation for the stable PA behavior. It could be speculated that since the vast majority of participants worked from home, they replaced time spent commuting with time spent engaging in exercise or other PA around the home.

As hypothesized, we found that, on average, there was a decrease in PA and an increase in screen time during the COVID-19 mandatory stay-at-home order; these findings are similar to what was found in an older, more racially homogenous sample of U.S. adults [28]; in French children age 11–17 years [27]; and in racially diverse U.S. high school students (decrease in PA only; screen time was not assessed) [26]. There are a multitude of factors that could explain the decrease in PA and increase in screen time, particularly related to barriers to accessing PA spaces and activities outside of the home and changes in recreational screen time. Namely, gyms and facilities dedicated to PA were closed as part of the stay-at-home order and may have disrupted the methods individuals normally used to engage in PA. Because many individuals were working from home, there was likely also a decrease in active transport, which is a common form of PA among adults [29]. Moreover, psychological changes due to the isolation and stress of COVID-19 may have led to a decrease in PA and an increase in screen time. Prior work has shown that social connection and loneliness predicted anxiety and depression during COVID-19, which has been shown to be associated with downstream behavioral effects [30]. Therefore, it is possible that the changes in social connection, loneliness, and subsequent changes in depression and anxiety [10] have led to decreased PA and increases in screen time. In an effort to increase connectivity, individuals may also be utilizing technology to connect more via phone or social media. Interestingly, we also found that there were some individuals who had increased PA and/or decreased screen time during COVID-19. It is possible that the mandatory stay-at-home order provided more recreational time that some devoted to increasing their PA. Alternatively, increased childcare, working at home, or increased family responsibilities for children, parents, or other family members may have limited recreational time, including screen time. This reduction in recreational time could have led to a proportion of individuals having a decrease in screen time.

Results of the present study shed light on important disparities in PA during the COVID-19 mandatory stay-at-home order. Identifying as Black or African American, identifying as being of mixed or other ethnicities/races, having low SES, and perceiving one’s neighborhood as unsafe during the day were each independently associated with lower PA. Notably, findings indicate that each of these sociodemographic and neighborhood characteristics explained PA levels during the mandatory stay-at-home order above and beyond the contribution of each of the other characteristics. Therefore, on average, individuals with more than one of these characteristics reported cumulatively lower levels of PA during the mandatory stay-at-home order. Moreover, these results accounted for PA levels prior to the COVID-19 pandemic, revealing that these results do not simply reflect pre-existing disparities, but rather, suggest disparities that may have been either introduced or magnified during the COVID-19 pandemic.

Lower levels of PA during an already highly stressful pandemic are particularly problematic for Black or African American and low SES communities because PA is effective in lowering the risk of chronic diseases such as obesity, type 2 diabetes, hypertension, and cardiovascular disease—all diseases that are commonly more prevalent in these populations [31]. COVID-19 deaths are higher in people with pre-existing chronic diseases; thus, communities of color (such as Black or African American) and lower SES are disproportionately affected by COVID-19 [32]. The higher rates of COVID-19, on top of the closure of non-essential facilities and activities, and the fear of contracting COVID-19 in open spaces may have affected communities of color and low SES communities differently, perhaps further contributing to the disparities seen in PA behaviors.

Our study suggests that feeling unsafe walking in one’s neighborhood was a determinant of PA and screen time during the pandemic stay-at-home order. Future research should consider the roles racism and discrimination may play in the perception of one’s environment, as findings from a Pew Research Center report about discrimination during COVID-19 indicate that 38% of Black or African Americans felt people acted like they were uncomfortable around them, 21% reported being targets of racial slurs or jokes, and 20% feared they might be threatened or physically attacked [33]. Further, our findings reported that Asian Americans and participants who perceived their neighborhood to be unsafe at night reported higher screen time during the COVID-19 mandatory stay-at-home order. Data collected during the COVID-19 outbreak document an increase of racist and discriminatory acts towards Asian Americans due to China being blamed for the cause of the COVID-19 outbreak [33]. Specifically, the earlier-mentioned Pew Research Center report found almost 40% of Asian Americans felt people acted like they were uncomfortable around them, 31% were targets of racial slurs or jokes and felt this increased since the COVID-19 outbreak, and 26% feared they might be threatened or physically attacked [33]. Almost a third of Asian Americans reported that discrimination took place in a public setting such as a park (15.5%), street (0.6%), or transit (15.2%) [34]. Racism and discrimination most likely play a role in individuals perceiving their neighborhood as unsafe, and subsequently, active transportation and other PA opportunities being hindered for socially minoritized groups, thus leading to increased recreational screen time indoors. At all times and not just during a pandemic, it is essential to keep this information in the forefront when intervening upon these health behaviors for communities of color because of its potential impact. However, more investigation is needed to fully understand the effects of COVID-19-specific discrimination on perceptions and behaviors of both Black or African Americans and Asian Americans.

### Strengths and Limitations

An important strength of this study is the longitudinal examination of PA and recreational screen use among a diverse population-based sample of emerging adults. Additionally, we were able to assess changes in PA and screen time pre- and post-mandatory stay-at-home orders, both categorically and linearly using previously validated survey measures, thereby identifying that there were individual differences in effects. Further, the longitudinal nature and analyses of the unique stay-at-home order allow us to identify the effects of this important public health initiative. However, while the stay-at-home period is an important period of time to examine for public health, our results may not be applicable to changes in PA or screen time throughout the COVID-19 pandemic, which warrants further research. Another major limitation of the present study is the use of self-reporting instead of objective measures to assess PA and recreational screen time. However, these measures are well-established and commonly used in survey methods with a high level of validity [18,19,20]. Additionally, the PA and recreational screen time questions collected during the pandemic did not specifically ask how COVID-19 affected these behaviors. Therefore, it cannot be ascertained whether the changes reported are specifically due to the pandemic. Lastly, the majority of the sampled individuals identified as female, which can affect the generalizability of the findings.

## 5. Conclusions

Findings indicate that stay-at-home policies may significantly impact PA levels and recreational screen time and that there is a need to reach populations at greatest risk for these changes in order to minimize adverse consequences. Neighborhood safety, coming from a low SES background, and being part of an ethnic/racial minoritized group were the strongest predictors of PA and screen time during the pandemic. These findings highlight potential mechanisms for health disparities that exist, particularly as a result of COVID-19. Post-COVID-19 interventions will be important to implement to ensure an increase in PA and a decrease in recreational screen time. Without attention to these behaviors, and the environmental conditions that influence these behaviors (e.g., neighborhood safety and opportunities for outdoor recreation), we can expect to see increases in adverse health outcomes and the widening of health disparities. Interventions aimed at increasing PA and decreasing screen time post-COVID-19 should focus on increasing neighborhood safety and focus on low SES and ethnic/racial minoritized populations.

## Figures and Tables

**Table 1:** Sample Characteristics during COVID-19 Mandatory Stay-at-Home Order (N = 218).

	Mean (SD)
Age (years)	24.6 (2.0)
	**% (n)**
Gender	
Male	28.4 (62)
Female	70.2 (153)
Other	1.4 (3)
Ethnicity/race ^1^	
White	36.4 (79)
Black/African American	14.3 (31)
Hispanic/Latinx	19.4 (42)
Asian American ^2^	19.4 (42)
Mixed/other	10.5 (23)
Socioeconomic status	
Low	30.3 (66)
Low-middle	16.1 (35)
Middle	18.3 (40)
High-middle	21.1 (46)
High	14.2 (31)
Temporarily laid off due to COVID-19	21.1 (46)
Has children in the home	18.8 (41)
Perceived lack of neighborhood safety	
During the day	11.5 (25)
At night	40.8 (89)

^1^ Ethnicity/race represents structurally racialized categories. The sample size for some variables may vary slightly from the total sample size due to missing responses. ^2^ Participants identifying as Asian American were primarily of Hmong background (71.4%).

**Table 2:** Changes in Physical Activity and Recreational Screen Time Adjusted for Pre-Pandemic Seasonal Effects ^1^.

	EAT 2018 (before COVID-19)	C-EAT (during COVID-19)	
	Estimated Marginal Mean (SE)		*p*
MVPA ^2^ (h/week)	4.7 (0.3)	3.5 (0.3)	<0.001
Total PA ^3^ (h/week)	7.9 (0.4)	5.8 (0.4)	<0.001
Recreational screen time (h/week)	26.5 (0.9)	29.4 (0.8)	0.003

^1^ Results from repeated ANCOVA analyses controlling for the month of the year during which the EAT 2018 survey was completed to account for potential pre-pandemic seasonal differences. ^2^ MVPA = moderate-to-vigorous physical activity. ^3^ PA = physical activity.

**Table 3:** Unadjusted Percentages of Participants who Experienced Decreases, No Changes, or Increases in Physical Activity and Recreational Screen Time from EAT 2018 (before COVID-19) to C-EAT (during COVID-19 mandatory stay-at-home order).

	Decrease	No Change	Increase
	% (n)		
MVPA ^1^	60.4 (131)	9.2 (20)	30.4 (66)
Total PA ^2^	58.8 (127)	8.8 (19)	32.4 (70)
Recreational screen time	29.6 (64)	21.8 (47)	48.6 (105)

^1^ MVPA = moderate-to-vigorous physical activity. ^2^ PA = physical activity.

**Table 4:** Separate Models Examining Predictors of Physical Activity and Recreational Screen Time during COVID-19 Mandatory Stay-at-Home Order ^1^.

Predictors (Examined in Separate Models)	MVPA ^2^		Total PA ^3^		Recreational Screen Time	
	*b*	*p*	*b*	*p*	*b*	*p*
Perceived lack of neighborhood safety						
During the day	**−1.59**	**0.04**	**−2.80**	**0.02**	1.71	0.45
At night	−0.98	0.05	−1.14	0.14	**4.01**	**0.007**
Age	−0.05	0.70	−0.14	0.44	−0.26	0.44
Gender						
Male (ref)	--	--	--	--	--	--
Female	0.37	0.51	0.24	0.78	1.15	0.48
Other	2.65	0.22	6.09	0.06	3.35	0.60
Ethnicity/race ^4^						
White (ref)	--	--	--	--	--	--
Black/African American	**−2.39**	**0.002**	**−3.16**	**0.007**	−1.86	0.42
Hispanic/Latinx	−1.12	0.10	−1.43	0.17	−0.87	0.67
Asian American	**−1.59**	**0.02**	**−2.37**	**0.02**	**4.87**	**0.02**
Mixed/other	**−1.79**	**0.03**	**−2.89**	**0.03**	4.02	0.11
Socioeconomic status						
Low	**−2.54**	**0.001**	**−4.04**	**<0.001**	0.41	0.86
Low-middle	**−2.20**	**0.01**	**−3.32**	**0.01**	−1.89	0.48
Middle	**−1.89**	**0.03**	**−3.47**	**0.008**	0.05	0.98
High-middle	−1.26	0.13	**−2.63**	**0.04**	2.95	0.24
High (ref)	--	--	--	--	--	--
Temporarily laid off due to COVID-19	−0.58	0.33	−1.11	0.22	2.83	0.11
Has children in the home	−0.82	0.20	−1.43	0.14	−1.90	0.31

^1^ All models were adjusted for pre-COVID-19 levels of physical activity or recreational screen time. Bold text indicates statistical significance at *p* < 0.05. The sample size for some variables may vary slightly from the total sample size due to missing responses. ^2^ MVPA = moderate-to-vigorous physical activity. ^3^ PA = physical activity. ^4^ Ethnic/racial categories were examined in these analyses as crude proxies for lifelong exposure to structural racism.

**Table 5:** Mutually Adjusted Models Examining Predictors of Physical Activity and Recreational Screen Time during COVID-19 Mandatory Stay-at-Home Order ^1^.

Predictors (Examined Simultaneously)	MVPA ^2^		Total PA ^3^		Recreational Screen Time	
	*b*	*p*	*b*	*p*	*b*	*p*
Perceived lack of neighborhood safety						
During the day	−1.50	0.07	**−2.75**	**0.03**	--	--
At night	--	--	--	--	**3.56**	**0.03**
Age	0.00	0.99	−0.07	0.71	−0.17	0.63
Gender						
Male (ref)	--	--	--	--	--	--
Female	0.63	0.26	0.64	0.44	0.36	0.83
Other	1.81	0.40	4.85	0.13	4.04	0.52
Ethnicity/race ^4^						
White (ref)	--	--	--	--	--	--
Black/African American	**−1.97**	**0.02**	**−2.57**	**0.04**	−0.95	0.69
Hispanic/Latinx	−0.74	0.34	−1.05	0.36	1.72	0.44
Asian American	−1.00	0.18	−1.56	0.17	**5.92**	**0.009**
Mixed/other	**−1.83**	**0.04**	**−3.08**	**0.02**	4.85	0.06
Socioeconomic status						
Low	**−1.77**	**0.04**	**−2.76**	**0.03**	−0.83	0.74
Low-middle	−1.17	0.22	−1.56	0.27	−3.34	0.22
Middle	−0.95	0.29	−1.99	0.14	−1.27	0.64
High-middle	−1.20	0.16	−2.41	0.06	3.40	0.17
High (ref)	--	--	--	--	--	--
Temporarily laid off due to COVID-19	−0.36	0.56	−0.80	0.39	3.19	0.08
Has children in the home	−0.63	0.36	−1.12	0.28	0.01	0.99

^1^ All models were adjusted for pre-COVID-19 levels of physical activity or recreational screen time. Bold text indicates statistical significance at *p* < 0.05. The sample size for some variables may vary slightly from the total sample size due to missing responses. ^2^ MVPA = moderate-to-vigorous physical activity. ^3^ PA = physical activity. ^4^ Ethnic/racial categories were examined in these analyses as crude proxies for lifelong exposure to structural racism.

## Data Availability

The data presented in this study are not publicly available but can be provided by senior author D.N.-S. in response to a reasonable request.
